# Experiences of aging in place in the United States: protocol for a systematic review and meta-ethnography of qualitative studies

**DOI:** 10.1186/s13643-018-0820-8

**Published:** 2018-10-06

**Authors:** Amy Rosenwohl-Mack, Karen Schumacher, Min-Lin Fang, Yoshimi Fukuoka

**Affiliations:** 1 Department of Social & Behavioral Sciences, School of Nursing, University of California, San Francisco (UCSF), CA USA; 2 Department of Physiological Nursing, School of Nursing, UCSF, CA, USA; 3 Education and Research Services, UCSF Library, UCSF, CA, USA; 4 Institute for Health & Aging, School of Nursing, UCSF, CA, USA

**Keywords:** Aging in place, Older adults, Qualitative research, Systematic review, Meta-ethnography, Living alone, Staying at home, Aging at home, Aging in community, Independent aging

## Abstract

**Background:**

By 2035, older adults will outnumber children for the first time in the United States (US). In light of its aging population, the US has supported services focused on enabling older adults to continue living in their current homes, a model commonly described as “aging in place.” The lived experience of aging in place is not well documented in existing systematic reviews. The aims of this systematic review are to synthesize and evaluate the existing qualitative evidence on experiences of aging in place in the US and identify knowledge gaps and directions for future studies.

**Methods:**

Six electronic bibliographic databases (PubMed, PsycINFO, CINAHL, Web of Science, EMBASE, and Sociological Abstracts) will be searched. Studies presenting qualitative data on the experiences of older adults currently aging in place in the US will be included. Covidence software will be used to screen studies and extract data. The Joanna Briggs Institute checklist for qualitative research will be used to assess quality and risk of bias of included studies. We will use meta-ethnography, following the method described by Noblit and Hare, to synthesize and evaluate the results of the included studies.

**Discussion:**

To the best of our knowledge, this is the first systematic review to integrate and synthesize the findings of qualitative studies of aging in place focusing on older adults in the US. The findings of this review will provide in-depth knowledge on lived experiences of aging in place and address important gaps in existing work.

**Systematic review registration:**

International Prospective Register of Systematic Reviews (PROSPERO): CRD42018102847

**Electronic supplementary material:**

The online version of this article (10.1186/s13643-018-0820-8) contains supplementary material, which is available to authorized users.

## Background

The United States (US) Census Bureau projects that older adults will outnumber children for the first time in the US by 2035 [1]. Although life expectancy has started to decline slightly over recent years, the average 65-year-old American can still expect to have another 20 years left to live [2]. The older adult population in the US is also becoming more diverse in terms of race/ethnicity, and older adults experience higher rates of chronic disease; in 2014, 81% of older adults in the US were living with two or more chronic diseases [3]. These rapid changes in population structure could have major implications for health and health care in the US, as well as for social and economic wellbeing.

In light of its aging population, the US federal government provides support for older adults to continue living in their current homes until the end of life, a model commonly described as “aging in place” [4]. This phrase covers a range of circumstances, but broadly refers to “the ability to live in one’s own home and community safely, independently, and comfortably, regardless of age, income, or ability level,” with relatively preserved independence compared to living in an institutional setting [5]. Although aging in place is assumed to be a cheaper option based on costs of care and lodging, it is difficult to calculate a true comparison given the hidden costs of family caregiving and unmet needs [6]. Regardless of cost, aging in place has wide public support, with 95% of those aged 50 or over reporting that they would prefer to remain in their current housing rather than entering institutional care [7]. However, among the subgroup of older adults who report that their home does not currently meet their physical needs, only 62% wish to age in place [7].

The lived experience of aging in place is not well documented in existing systematic reviews. Although there are several published systematic reviews examining the provision of specific services for community-dwelling older adults, such as home health care or telecare [8, 9], only two systematic reviews focus specifically on aspects of aging in place: a systematic review of quantitative studies on cost effectiveness of aging in place [10], and a systematic review of factors influencing acceptance of technology for aging in place [11]. The authors of the cost effectiveness review [10] found the existing evidence to be low quality and limited by inconsistencies in measurement, so they were unable to draw any robust conclusions. The systematic review of technology acceptance identified concerns about finances and privacy, maintaining control and independence, and fear of stigmatization and institutionalization [11]. Although these two systematic reviews may contribute to our understanding of aging in place, each focuses on one isolated element of the lived experience. In contrast, a brief survey of recently published primary research on aging in place reveals a complex and diverse phenomenon that extends beyond issues relating to cost and technology. For example, researchers have described the experiences of older immigrants aging “out of place” and perspectives of lesbian, gay, bisexual, transgender, and queer (LGBTQ) older adults on aging in place in potentially hostile communities [12, 13].

A more holistic synthesis of older adults’ experiences is needed to evaluate the tensions and complexities that may be characteristic of aging in place in diverse circumstances. Moreover, existing reviews consider evidence across multiple countries and health and social care systems, without attention to the contextual and cultural factors that may inform differences in experiences or that situate aging in place in particular health and long-term care systems. The lack of an existing systematic review of qualitative studies on aging in place limits our understanding of the experiences of older adults. A synthesis of the evidence could identify contextual factors promoting aging in place, helping providers to design more appropriate services and to recognize those older adults who may face particular challenges in the US. The proposed systematic review aims to address this significant gap in the existing literature.

People who are aging in place are often invisible to the rest of the population, particularly if their mobility or resources restrict their ability to engage in the local community. Current debates around aging in place center on individuals’ reported preferences for the future, rather than considering the current reality of those already aging in place. With increasing levels of loneliness reported among older people, as well as our growing understanding of the seriousness of the health consequences associated with social isolation [14], a systematic review of the available qualitative evidence is needed to ensure the voices of those with lived experience of aging in place in the US are heard. The aims of this systematic review are to synthesize and evaluate the existing qualitative evidence on experiences of aging in place in the US and to identify knowledge gaps and directions for future studies.

## Methods

This systematic review has been registered with the International Prospective Register of Systematic Reviews (PROSPERO): registration number CRD42018102847.

The Preferred Reporting Items for Systematic Review and Meta-Analysis Protocols (PRISMA-P) statement [15] has been used in the preparation of this protocol, and the PRISMA-P 2015 Checklist is included (see Additional file 1).

Meta-ethnography will be used to synthesize and evaluate the results of the included studies, following the method outlined by Noblit and Hare [16] with reference to Schutz’s conceptualization of first- and second-order constructs [17, 18]. This approach was selected in light of its suitability for studying complex social phenomena and experiences, specifically through generating new interpretations and theoretical models (i.e., going beyond the categorization or aggregation of existing findings) [16, 17]. Noblit and Hare’s methodology is comprised of seven phases: (1) getting started, (2) deciding what is relevant to the initial interest, (3) reading the studies, (4) determining how the studies are related, (5) translating the studies into one another, (6) synthesizing translations, and (7) expressing the synthesis.

### Phase 1: Getting started

AR-M identified the focus of the meta-ethnography, and a review of qualitative research was deemed the most appropriate approach to gain a rich understanding of the lived experience of aging in place.

## Information sources and search strategy

In collaboration with a medical librarian (MF), a systematic search strategy for six electronic databases (PubMed, Embase, PsycINFO, CINAHL, Web of Science, and Sociological Abstracts) was designed using a combination of MeSH/Emtree terms and various keywords to identify peer-reviewed studies related to aging in place. No date limits will be applied to the searches, in order to evaluate the emergence of the concept of aging in place in the literature over time. Appendix provides detailed sample search strategies. In addition, Web of Science will be used to find citing, cited, and relevant references of studies selected for inclusion. Reference lists of related published qualitative syntheses will also be searched, with hand searching of relevant journals used to locate additional studies.

### Phase 2: Deciding what is relevant to the initial interest

The search strategies were developed using broad initial iterative searches to explore how the relevant literature is coded and described in the databases used. For example, there is no MeSH term for “aging in place” in PubMed, and relevant papers are often not specifically tagged as referring to older adults, presumably because the concept of aging in place already contains this concept. Initial exploratory searches informed the decision to expand the search terms beyond direct variations on “aging in place” to include other phrases relating to living and aging in community settings; although this choice is likely to increase the number of studies to be screened, it is designed to improve the sensitivity of the search strategy.

The SPIDER tool [19] was used to structure the search terms and eligibility criteria (see Table 1). In constructing the search strategies, design, evaluation, and research type were combined using the Boolean operator OR instead of AND, since the indexing of qualitative research in databases is highly variable. There is no inclusion/exclusion criterion relating to age, since chronological age can be a poor marker for the personal lived experience of aging [20]; the age range of participants in the included studies will form part of the review findings.

**Table 1:** SPIDER table of study inclusion and exclusion criteria

	Inclusion criteria	Exclusion criteria
Sample	• Community-dwelling older adults living in the US	• People living in institutional settings (e.g., nursing home or age-segregated supportive housing) • People who have already relocated or decided to relocate to an institutional setting
Phenomenon of interest	• Studies about aging in place (e.g., intending to live in own home until end of life)	• Studies examining only the perspectives of staff or caregivers • Studies focusing on appraisal of local neighborhood environment
Design	• Qualitative or mixed-methods studies reporting primary qualitative data (e.g., through interviews, focus groups, participant observation)	• Studies reporting only quantitative data (e.g., cross-sectional, case-control, cohort studies, clinical trials)
Evaluation	• Qualitative analysis of experiences, feelings, views, opinions, and plans	• Evaluation using quantitative methods only
Research type	• Peer-reviewed journal articles • Full text available in English	• Systematic reviews, protocols, theoretical work, editorials, opinion pieces, and dissertations

## Study selection and data management process

A flow diagram following the PRISMA guidelines for reporting systematic reviews will be used to illustrate the selection processes and results [21]. Initially, all retrieved studies will be imported into Endnote reference management software [22] to remove duplicates, then the remaining citations will be uploaded to Covidence software [23] which is recommended by Cochrane for use by its systematic review authors. Two independent reviewers will screen study titles and abstracts against the inclusion and exclusion criteria, followed by full-text review of remaining studies to determine eligibility. The two reviewers will discuss any discrepancies; if they cannot reach an agreement, a third reviewer will make a final decision.

## Quality assessment

The question of how to assess quality and risk of bias in qualitative research has been the subject of ongoing debate, with some scholars arguing that it is undesirable or even impossible to establish a set of universal standards by which to judge qualitative research [24]. However, although it is clearly inappropriate to apply positivist criteria created for quantitative research, there is nonetheless value in taking a systematic approach to the evaluation of qualitative studies [25], particularly when seeking to evaluate the strength of the evidence gathered by a systematic review. In this review, the Joanna Briggs Institute Checklist for Qualitative Research will be used to critically appraise the quality and risk of bias of each included paper; this tool was chosen as it is specifically designed for the evaluation of congruity in systematic reviews of qualitative studies [26, 27]. The checklist includes 10 questions, focusing on consistency of theory and methods, reflexivity and positionality, and ethical practice, and we added two fields after pilot testing: relevance to the synthesis and overall quality assessment. Following Dixon-Woods and colleagues [28], we will assign each paper to one of four categories based on the results of the checklist: key paper; satisfactory paper; irrelevant to the synthesis; and fatally flawed. Two members of the research team will independently assess the quality of each included study using the checklist; any differences of opinion will be resolved by discussion and involvement of a third researcher as required. Studies will not be excluded based on quality alone, but this information will be used to weight the contribution of their results to the meta-ethnographic synthesis [17]. An evaluation of the overall quality of the literature reviewed will also form part of the study findings, in terms of any research gaps or weaknesses identified; we will use the Confidence in the Evidence from Reviews of Qualitative Research (CERQual) assessment approach to evaluate our confidence in our review findings by creating a summary of qualitative findings table [29].

## Data items and data collection process

One reviewer will use Covidence software [23] to extract study characteristics, including (but not limited to) description of references (e.g., authors, publication date, aims), socio-demographics (e.g., location, mean age, gender, race/ethnicity), method and approach (e.g., interviews, focus groups), theoretical or conceptual framework used in the study (e.g., activity theory), and key themes reported in the study. If aging in place is defined in the study, we will record the definition presented. Data on the lived experience of aging in place in each study will be collected in the next phase of our review through meta-ethnography. A second reviewer will cross-check the accuracy of the extracted data. The data items to be extracted will be pilot tested using a template spreadsheet and a sample of potential studies obtained through the initial iterative search process.

## Synthesis of qualitative results

### Phase 3: Reading the studies

Our synthesis will begin with a process of repeated close reading of the included studies and memo writing on our initial impressions of the characterization of lived experiences of aging in place [16]. The full text of each included study will be imported into NVivo qualitative data analysis software [30] to facilitate coding and comparison. Throughout the process of meta-ethnographic analysis and synthesis, two reviewers will initially complete coding and data extraction independently, before working together to discuss differences and congruences in their analyses, and to identify and synthesize emerging themes.

### Phase 4: Determining how the studies are related

The first step will involve the coding of second-order constructs from the results section of each included study, consisting of the original researchers’ interpretation of their participants’ words [17, 18]. We will also extract first-order constructs—raw data representing participants’ experiences and interpretations—to illustrate each second-order construct [18].

### Phase 5: Translating the studies into one another

The extracted first- and second-order constructs will be compared across studies to identify similarities and differences, which will inform the “translation” process described by Noblit and Hare [16]. The process of deriving themes will therefore be inductive—driven by the data rather than based on any a priori framework.

### Phase 6: Synthesizing translations

As we translate the extracted constructs in this way, we will develop third-order constructs: our own interpretations of the second-order constructs in the included studies. Although the steps of the meta-ethnographic synthesis are presented in order here for clarity, the process is not strictly linear but will involve iteration and re-evaluation.

### Phase 7: Expressing the synthesis

Tables and figures will be used for the synthesis and evaluation of the reviewed studies, following the methodology outlined by Malpass et al. [31], alongside a narrative account of the overarching themes and processes identified.

## Discussion

### Limitations and strengths

To the best of our knowledge, this is the first systematic review to integrate and synthesize the findings of qualitative studies of aging in place focusing on older adults in the US. Although the findings of this systematic review may not be generalizable to aging in place in other countries, we believe it is important to focus on the US because of its specific demographic and political context. We also recognize that the value of aging in place is culturally determined and informed by differences in economic and social structures, among other factors [32]. Therefore, it would be challenging to draw meaningful conclusions from a set of studies conducted in countries involving different health systems and settings. The findings of this systematic review will inform a necessary and timely discussion about what it means to age in place in the US.

The lack of a single definition of “aging in place,” even within the US, could present a challenge to our review methodology. We decided to design our search strategy to include a range of related phrases, sacrificing some specificity but maximizing sensitivity in our search process. In collaboration with an experienced librarian, our inclusion and exclusion criteria have been carefully structured to focus in on the phenomenon of intending to remain in one’s home for as long as possible, and three reviewers will independently screen and select studies for inclusion to ensure this process is robust. This systematic review was designed following established protocols to maximize rigor and transparency; it addresses a vital gap in the existing literature on lived experiences of older adults in the US and will highlight key areas to be considered in future research and policy.

### Additional file

Additional file 1: PRISMA-P 2015 Checklist: Experiences of aging in place in the United States: protocol for a systematic review and meta-ethnography of qualitative studies. (DOCX 34 kb)

## Appendix

### Sample search strategies for PubMed and EMBASE

PubMed:

**Table:**

**#**	Query
1	(“aging in place”[Title/Abstract] OR “aging in place”[Title/Abstract] OR “age in place”[Title/Abstract] OR “aging-in-place”[Title/Abstract] OR “aging-in-place”[Title/Abstract] OR “aging at home”[Title/Abstract] OR “aging at home”[Title/Abstract] OR “growing older at home”[Title/Abstract] OR “growing old at home”[Title/Abstract] OR “aging in communities”[Title/Abstract] OR “aging in community”[Title/Abstract] OR “aging in communities”[Title/Abstract] OR “aging in community”[Title/Abstract])
2	(old[Title/Abstract] OR older[Title/Abstract] OR senior[Title/Abstract] OR seniors[Title/Abstract] OR elderly[Title/Abstract] OR elder[Title/Abstract] OR elders [Title/Abstract] OR geriatric[Title/Abstract] OR “Aged”[MeSH])
3	(“stay at home”[Title/Abstract] OR “staying at home”[Title/Abstract] OR “live at home”[Title/Abstract] OR “living at home”[Title/Abstract] OR “living alone”[Title/Abstract] OR “meaning of home”[Title/Abstract] OR “concept of home”[Title/Abstract] OR “remaining at home”[Title/Abstract] OR “remain at home”[Title/Abstract])
4	(“Qualitative Research”[Mesh]) OR qualitative[Title/Abstract] OR “mixed method” [Title/Abstract] OR “mixed methods”[Title/Abstract] OR “focus group”[Title/Abstract] OR “focus groups”[Title/Abstract] OR interview[Title/Abstract] OR interviews[Title/Abstract] OR interviewing[Title/Abstract] OR interviewed[Title/Abstract] OR ethnography[Title/Abstract] OR ethnographic[Title/Abstract] OR phenomenology[Title/Abstract] OR phenomenological[Title/Abstract] OR “grounded theory”[Title/Abstract] OR “case study”[Title/Abstract] OR “constant comparative”[Title/Abstract] OR “constant comparison”[Title/Abstract] OR “content analysis”[Title/Abstract] OR “discourse analysis”[Title/Abstract] OR “narrative”[Title/Abstract] OR “participant observation”[Title/Abstract] OR “field study”[Title/Abstract] OR “field studies”[Title/Abstract] OR “concept analysis”[Title/Abstract] OR view[Title] OR views[Title] OR experience[Title] OR experiences[Title] OR feel*[Title] OR know*[Title] OR opinion*[Title] OR belief[Title] OR beliefs[Title] OR descriptive[Title] OR expectation[Title] OR expectations[Title] OR perception[Title] OR perceptions[Title])
5	#2 AND #3
6	#1 OR #5
7	#4 AND #6

EMBASE:

**Table:**

**#**	Query
1	‘aging in place’:ab,ti OR ‘aging in place’:ab,ti OR ‘age in place’:ab,ti OR ‘aging-in-place’:ab,ti OR ‘aging-in-place’:ab,ti OR ‘aging at home’:ab,ti OR ‘aging at home’:ab,ti OR ‘growing older at home’:ab,ti OR ‘growing old at home’:ab,ti OR ‘aging in communities’:ab,ti OR ‘aging in community’:ab,ti OR ‘aging in communities’:ab,ti OR ‘aging in community’:ab,ti
2	‘aged’/mj
3	‘old’:ab,ti OR ‘older’:ab,ti OR ‘senior’:ab,ti OR ‘seniors’:ab,ti OR ‘elderly’:ab,ti OR ‘elder’:ab,ti OR ‘elders’:ab,ti OR ‘geriatric’:ab,ti
4	‘stay at home’:ab,ti OR ‘staying at home’:ab,ti OR ‘live at home’:ab,ti OR ‘living at home’:ab,ti OR ‘living alone’:ab,ti OR ‘meaning of home’:ab,ti OR ‘concept of home’:ab,ti OR ‘remaining at home’:ab,ti OR ‘remain at home’:ab,ti
5	‘qualitative research’/mj OR ‘qualitative’:ab,ti OR ‘mixed method’:ab,ti OR ‘mixed methods’:ab,ti OR ‘focus group’:ab,ti OR ‘focus groups’:ab,ti OR ‘interview’:ab,ti OR ‘interviews’:ab,ti OR ‘interviewing’:ab,ti OR ‘interviewed’:ab,ti OR ‘ethnography’:ab,ti OR ‘ethnographic’:ab,ti OR ‘phenomenology’:ab,ti OR ‘phenomenological’:ab,ti OR ‘grounded theory’:ab,ti OR ‘case study’:ab,ti OR ‘constant comparative’:ab,ti OR ‘constant comparison’:ab,ti OR ‘content analysis’:ab,ti OR ‘discourse analysis’:ab,ti OR ‘narrative’:ab,ti OR ‘participant observation’:ab,ti OR ‘field study’:ab,ti OR ‘field studies’:ab,ti OR ‘concept analysis’:ab,ti OR ‘view’:ti OR ‘views’:ti OR ‘experience’:ti OR ‘experiences’:ti OR ‘feel*’:ti OR ‘know*’:ti OR ‘opinion*’:ti OR ‘belief’:ti OR ‘beliefs’:ti OR ‘descriptive’:ti OR ‘expectation’:ti OR ‘expectations’:ti OR ‘perception’:ti OR ‘perceptions’:ti
6	#2 OR #3
7	#4 AND #6
8	#1 OR #7
9	#5 AND #8

## Abbreviations

CERQual: Confidence in the Evidence from Reviews of Qualitative Research
LGBTQ: Lesbian, Gay, Bisexual, Transgender, and Queer
PRISMA: Preferred Reporting Items for Systematic Reviews and Meta-Analyses
PROSPERO: International Prospective Register of Systematic Reviews
SPIDER: Sample, Phenomenon of Interest, Design, Evaluation, Research type
US: United States
